# Far-Red Absorbing LHCII Incorporating Chlorophyll *d* Preserves Photoprotective Carotenoid Triplet–Triplet Energy Transfer Pathways

**DOI:** 10.1021/acs.jpclett.4c03463

**Published:** 2025-02-10

**Authors:** Niccolò Cianfarani, Andrea Calcinoni, Alessandro Agostini, Eduard Elias, Marco Bortolus, Roberta Croce, Donatella Carbonera

**Affiliations:** † Department of Chemical Sciences, University of Padova, via Marzolo 1, 35131 Padova, Italy; ‡ Biophysics of Photosynthesis, Department of Physics and Astronomy, Faculty of Science, Vrije Universiteit Amsterdam and LaserLaB Amsterdam, De Boelelaan 1100, 1081 HZ Amsterdam, The Netherlands

## Abstract

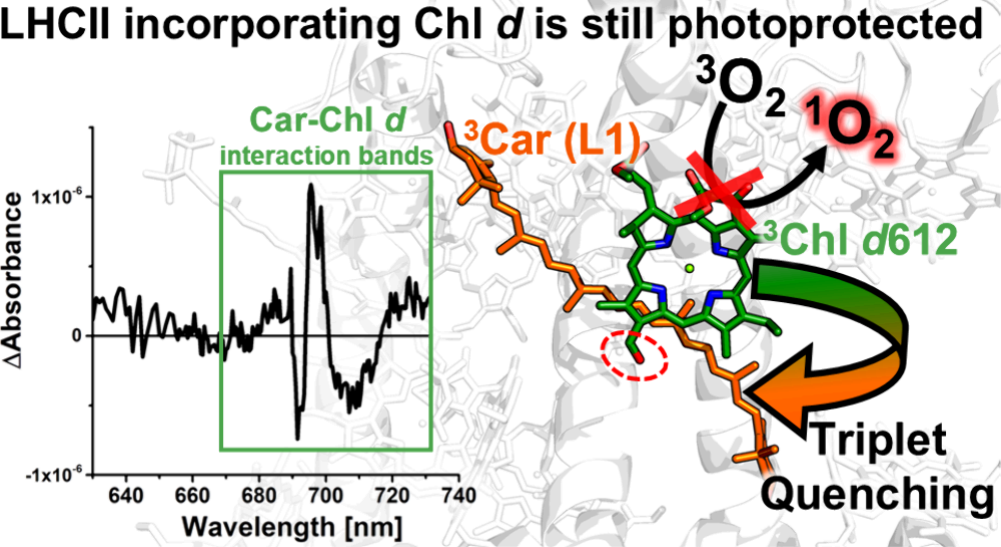

Chlorophyll *d* (Chl *d*) can be successfully introduced in reconstituted LHCII with minimal interference with the energy equilibration processes within the complex, thereby facilitating the development of plant light-harvesting complexes (LHCs) with enhanced capabilities for light absorption in the far-red spectrum. In this study, we address whether Chl *d* introduction affects LHCII’s ability to protect itself from photo-oxidation, a crucial point for successfully exploiting modified complexes to extend light harvesting in plants. Here we focus on incorporating Chl *d* into Lhcb1 (the monomeric unit of LHCII), specifically studying the Chl triplet quenching by carotenoids using time-resolved electron paramagnetic resonance (TR-EPR) and optically detected magnetic resonance (ODMR). We also characterize the A2 mutant of LHCII, in which the Chl 612 is removed, to assist in determining the triplet quenching sites on the Lhcb1 complex reconstituted with Chl *d*. We found that far-red absorbing LHCII incorporating Chl *d* maintains the efficiency of the photoprotective process.

LHCII is the primary light-harvesting complex of plants, absorbing photons in the visible spectrum up to 700 nm. This spectral limitation is particularly significant for leaves in densely packed canopies, especially at the bottom where only filtered light penetrates. As it turns out, the light reaching the lower levels of a canopy is rich in far-red and near-infrared photons (λ > 700 nm), which cannot be used for photosynthesis. This has suggested the idea of trying to expand the absorption cross-section of plants toward these spectral regions to potentially optimize crop growth and increase biomass yield.^1−4^ One approach to achieve this goal has been inspired by some cyanobacteria that are capable of far-red light absorption due to the incorporation of the red-shifted Chls *d* and/or *f* into their photosystems.^5−7^

Chl *d* serves as the primary light-absorbing pigment in *Acaryochloris marina*, which utilizes far-red photons to conduct photosynthetic processes with an efficiency comparable to that observed in plants. The biosynthesis of Chl *d* in *A. marina in vivo* is hypothesized to proceed through a single enzymatic reaction utilizing Chl *a* as a substrate,^8^ thereby potentially enabling the development of plants capable of synthesizing Chl *d* once the enzyme responsible for this transformation is identified. The capacity to synthesize Chl *f* and far-red-shifted phycobiliproteins can be reversibly triggered in various cyanobacteria cultivated under far-red light-enriched environments,^9,10^ a phenomenon named Far-Red Light Photoacclimation (FaRLiP).

It has been estimated that integrating cyanobacterial Chls *d* or *f* into plant LHCII has the potential to achieve a 19% increase in photosynthetic efficiency.^1^ Previous investigations have demonstrated that Chl *d* can be successfully incorporated into reconstituted LHCII, with minimal disturbance to the excited state decay or energy equilibration processes within the complex, thereby enabling the design of plant LHCs capable of improved light harvesting in the far-red spectrum,^11^ with the goal of achieving a “smart canopy”,^3^*i.e.* crops characterized by an increased far-red light absorption in the lower part of the canopy. Recently, the PSI antenna Lhca4 was reconstituted with far-red absorbing chlorophylls (Chls *d* and *f*).^12^ Chl *f* was incorporated with good affinity only in a few sites, whereas Chl *d* can bind to the sites responsible for the red-forms of the complex, thereby inducing a further red-shift of 20 nm.^12^

It is well established that photoprotection by carotenoids in light-harvesting complexes (LHCs) is a crucial mechanism that ensures the efficiency and longevity of photosynthetic organisms. The ubiquity of carotenoids in photosynthetic organisms across different taxa underscores their evolutionary importance. The conservation of carotenoid-based photoprotection mechanisms highlights their critical role in the success and diversification of photosynthetic life. This process is essential for maintaining the delicate balance between light harvesting and photoprotection, allowing plants and other photosynthetic organisms to thrive in diverse light environments. While efficient light harvesting is necessary for photosynthesis, in conditions of excessive light harmful reactive oxygen species (ROS), particularly singlet oxygen (^1^O_2_), can be formed. Similarly to Chl *a*, also Chl *d* in its triplet state can effectively photosensitize singlet oxygen.^13^

LHCs from plants and algae represent the first line of defense against ^1^O_2_ formation during photosystem overexcitation when Chl triplets are formed. Within the LHCs, carotenoids quench Chl triplets through triplet–triplet energy transfer (TTET).^14,15^ Within LHCs, this photoprotective mechanism has been identified to involve the two central carotenoids situated at the L1 and L2 sites in various LHCs from different organisms.^16−21^ The cluster of conserved pigments encompassing the L1 and L2 sites seems to represent a conserved structural motif of critical importance to the photoprotective apparatus of this protein class,^17^ with the active pairs being Chl *a*612/L1 and Chl *a*603/L2 (see Figure 1). The structural requirements for the interaction between carotenoids and chlorophylls, are critical for efficient photoprotection.^22^ In this work, we focus on incorporating Chl *d* into Lhcb1 (the monomeric unit of LHCII), specifically studying the triplet states of the complex, using Time-Resolved Electron Paramagnetic Resonance (TR-EPR) and Optically Detected Magnetic Resonance (ODMR), two powerful spectroscopic techniques for analyzing triplet states.^23,24^ We aim to fully understand the photophysical functioning of the antenna with exogenous red-shifted chlorophylls and determine if their introduction affects LHCII’s ability to protect itself from photo-oxidation, a crucial point for successfully exploiting modified complexes to extend light harvesting in plants.

**Figure 1:**
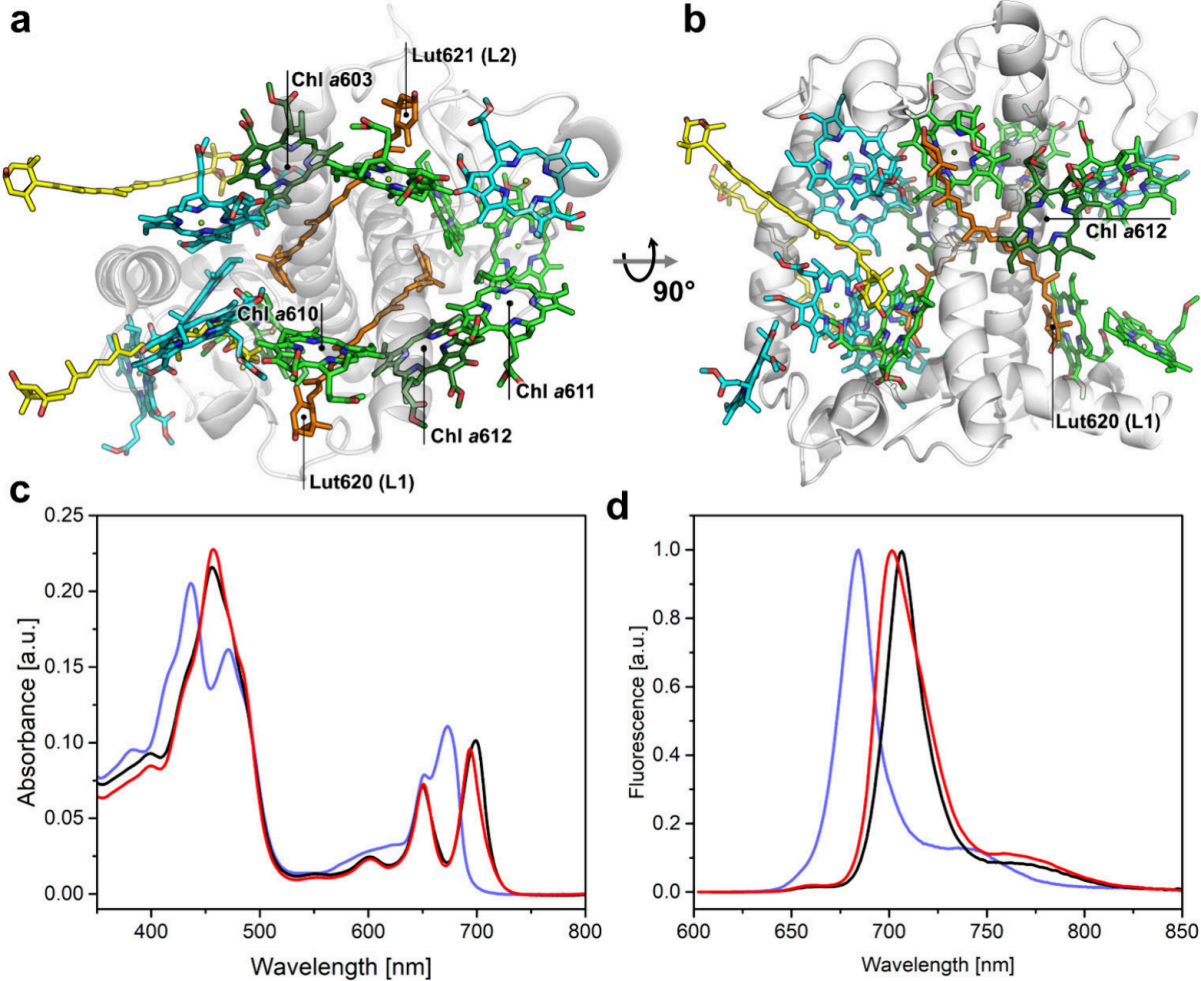
Lhcb-*ab* WT crystallographic structure:^26^ (a) stromal and (b) membrane view. The pigments are shown in stick representation, the protein scaffold in white cartoon representation. Green, Chls *a*; dark green, Chls *a* in sites *a*603 and *a*612; cyan, Chls *b*; orange, luteins at sites L1 (Lut620) and L2 (Lut621); yellow, neoxanthin and violaxanthin. RT (c) absorption and (d) fluorescence (λ_ex_ = 500 nm) of Lhcb-*db* WT (black line), Lhcb-*db* A2 (red line), and Lhcb-*ab* (blue line); the absorption spectra are normalized on the Q_*y*_ band (range λ = 630–750 nm) considering both the relative oscillator strength of the Chls (*a*:*b*:*d* = 1.0:0.67:1.2) and the pigment composition of each complex.

Alongside LHCII WT, we also characterize the A2 mutant in which the Chl 612 binding site is removed^25^ by means of a N183L mutation directed at preventing its magnesium-ligation (we will refer to the Chl-binding sites following Liu et al.^26^ throughout the manuscript. Note that the 612 binding site corresponds to the A2 site in Khulbrandt et al.^27^). Chl 612 is at the heart of the cluster that originates the lowest energy exciton state in the native complex containing Chls *a* and *b*.^28−30^ It is also the main site of the triplet quenching.^16^ Therefore, A2 mutant is expected to assist in determining the triplet quenching sites, similarly to previous work carried out on the native (containing Chls *a* and *b*) LHCII complex.^16^

In this study, we have reconstituted the Lhcb1 apo-proteins using a pigment mixture with a Chl *d*/*b* ratio of 3:1, achieving a Chl *d*/*b* ratio within the complex that closely approximates the Chl *a*/*b* ratio observed in native Lhcb1, which are 1.2 and 1.4 respectively, as documented in Table 1. It should be noted that prior data presented by Elias et al.^11^ were derived from a complex with a comparatively lower proportion of Chl *d* (refer to Table 1). The pigment analysis detailed in Table 1 indicates that the A2 mutant is deficient by one Chl *d* in comparison to the wild type (WT).

**Table 1:** Pigment Composition of the Lhcb-*db* Complexes Has Been Normalized to the Sum of Lut and Vio (Lut + Vio = 2)

Pigments	Lhcb-*db* WT	Lhcb-*db* A2	Lhcb-*ab* WT^a^	Lhcb-*ab* A2^a^
Chl *a*	-	-	7	5.5
Chl *d*	6.5 ± 0.3	5.6 ± 0.1	-	-
Chl *b*	5.5 ± 0.3	5.4 ± 0.1	5	3.8
Chl *d*(*a*)/*b*	1.2 ± 0.1	1.0 ± 0.0	1.4	1.4
Chl/Car	4.1 ± 0.2	3.9 ± 0.1	3.9	3.6
Lut	1.8 ± 0.0	1.7 ± 0.0	1.7	1.7
Vio	0.2 ± 0.0	0.3 ± 0.0	0.3	0.1
Neo	0.9 ± 0.1	0.8 ± 0.1	1.1	0.8

a Remelli et al.^25^

Figure 1c shows the absorption spectrum of the Lhcb-*db* complexes prepared in this work compared to that of Lhcb-*ab* WT. The absorption maximum of Lhcb-*db* WT is shifted from 672 to 699 nm, while the spectra have similar features in the Chl *b* Q_*y*_ absorption region (∼650 nm). The B_*x*_/B_*y*_ (Soret) Chl *d* bands in Lhcb-*db* are also shifted in energy compared to those of Lhcb-*ab*. In the case of the A2 mutant, a loss of a red contribution is visible in the Q_*y*_ peak of Chl *d* (702 nm), as anticipated due to the disruption of the redmost exciton cluster (composed of Chls 610–611–612 in WT). This is also confirmed by the corresponding Circular Dichroism spectra (Figure S1).

The emission spectra reported in Figure 1d show that the terminal emitter in Lhcb-*db* is either a single or cluster of Chl *d*. The small emission peak at ∼655 nm indicates the presence of a very low amount of unconnected Chl *b*. The high efficiency of the energy transfer process among the pigments in Lhcb-*db* is demonstrated by comparing the 1-T (Transmittance) with the excitation spectra of the complex (see Supporting Figure S2). The broadening of the emission profile of the A2 mutant is also an indication of the disruption of the lowest energy exciton. In the WT, the emission from this exciton is expected to be narrower due to exciton delocalization, whereas in the case of the A2 mutant, emission can originate from multiple sites, broadening the emission profile.^28^

In a prior study,^11^ some of us demonstrated that Chl *d* is functionally incorporated into LHCII, preserving the functional architecture of the native complex as well as the high light-harvesting efficiency characteristic of LHCII. Moreover, the terminal emitter seems to be conserved, a condition important for efficient excitation energy transfer to other subunits of the PSII supercomplexes and its use for photochemistry. It was also found that LHCII-*db* maintains an excitonic architecture comprising Chl-Chl interactions responsible for the red-most features of the absorption spectrum. In particular, the red-cluster still originates from the Chl 611–Chl 612 pair.

In native LHCII, Chls *a*612 and *a*603 are involved in triplet–triplet energy transfer (TTET) to Lut620 and Lut621, respectively.^16,19^ In principle, the complete substitution of Chl *a* by Chl *d* could alter the efficiency of this optimized mechanism. To fully characterize the TTET process in Chl *d* reconstituted samples, we employed time-resolved and pulse EPR, and ODMR.

Figure 2a-b shows the TR-EPR spectra of Lhcb-*db*, WT and mutant A2 at a DAF = 0.8 μs (Delay After the laser Flash, the complete time evolution of the signal is available in Supporting Figure S3). The spectra show the presence of two principal triplet populations, attributable to ^3^Car and ^3^Chl *d*. The distinct zero-field splitting (ZFS) parameters associated with these two chromophore triplets lead to the assignment of the peaks situated nearer the center of the spectra (approximately at 338 and 350 mT) to the low and high field Y transitions of ^3^Chl *d* (refer to Supporting Figure S4 for the TR-EPR spectrum of ^3^Chl *d* in micelles). Conversely, the external features (around 305 and 385 mT) can be attributed to the low and high field Z transitions of ^3^Car. In the central region of the spectrum, a substantial overlap between the triplet spectra of chlorophylls and carotenoids is observed, which is a frequent occurrence in LHCs TR-EPR spectra.^16,17,19,21,31−33^ The presence of residual ^3^Chl *d* states in Lhcb-*db*, *i.e*. ^3^Chls not able to transfer their energy to Cars, is a common feature observed also in native^19^ and recombinant^16^ LHCII, suggesting a common behavior in both complexes. The Chl to Car TTET process is generally completed in subnanosecond time scales; therefore, the polarization of the ^3^Car observed at short DAF can be considered the one acquired from the ^3^Chl during TTET since the triplet sublevel polarization is still unmodified by the anisotropic relaxation.

**Figure 2:**
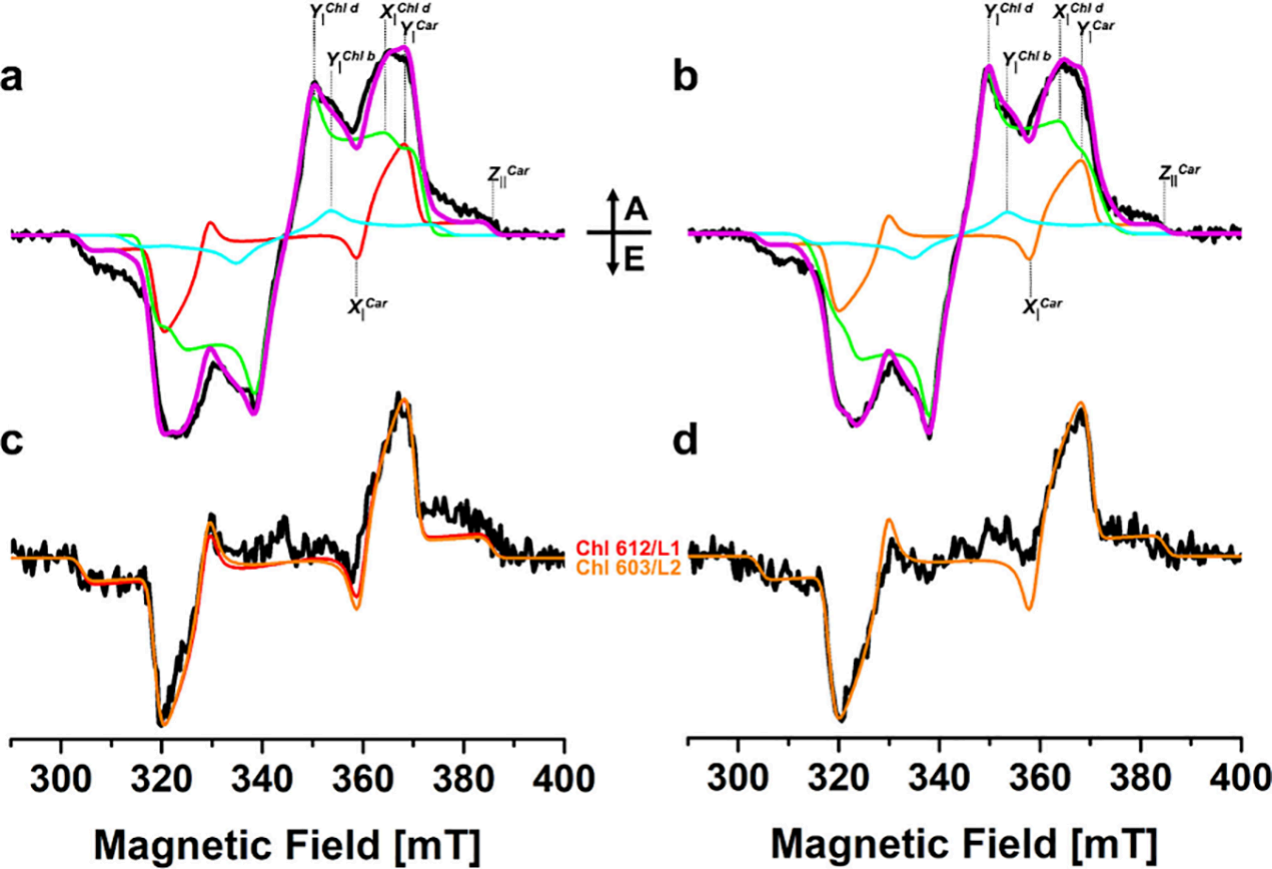
X-band TR-EPR (a, b) and ^3^Car FS-ESE (c, d) spectra of Lhcb-*db* WT (a, c) and A2 (b, d). Glycerol/water buffer (66% *v*/*v*) glass; *T*° = 80 K; λ_ex_ = 532 nm; *A* = absorption, *E* = emission. The TR-EPR traces are the average of the spectra in the 0.7–0.9 μs time range after the laser flash (see Supporting Figure S3). The FS-ESE spectra of Lhcb-*db* have been corrected for the residual presence of ^3^Chl *d* by subtracting a corresponding spectrum of ^3^Chl *d* in Tx-100 micelles (see Supporting Figure S5), and the difference corresponds to the “pure” ^3^Car spectrum (panels c and d, black lines). The simulations of the ^3^Car spectra for L1 or L2 (red and orange lines, respectively) are obtained using triplet sublevel polarizations derived by a TTET starting from the triplet state of Chls 612 or 603, respectively, calculated on the basis of atomic coordinates for the acceptor–donor pairs derived from the crystallographic structure^26^ and an initial donor ^3^Chl *d* polarization (P_*x*_:P_*y*_:P_*z*_ = 0.36:0.45:0.19, see Supporting Figure S4). The result is a ^3^Car polarization of (P_*x*_:P_*y*_:P_*z*_ = 0.42:0.20:0.38) for 612/L1 and (P_*x*_:P_*y*_:P_*z*_ = 0.42:0.21:0.37) for 603/L2. The global Lhcb-*db* TR-EPR spectra (purple lines) are the sum of the individual lineshapes of ^3^Car (red and orange lines), ^3^Chl *d* (green lines), and ^3^Chl *b* (cyan lines); the proportion of each component is indicated in the spectra. All simulation parameters are reported in Table 2. Canonical transitions are indicated for the high-field half of the spectra. The spectra have been vertically translated for clarity.

Field-swept electron spin echo (FS-ESE) triplet spectra of the three samples at short DAF were also collected since we previously demonstrated that in these experiments the line shape contribution of unquenced ^3^Chl is largely suppressed^17,19^ due to the strong anisotropic relaxations of porphyrins,^34^ leaving the contribution of ^3^Car easily discernible (Figure S5). The polarization pattern of ^3^Car, *eeaeaa* in both complexes (Figure 2c-d), is very similar to that of Lhcb-*ab*.^16^ The spectral shapes of ^3^Car were well reproduced by calculations performed on the basis of the atomic coordinates for the acceptor–donor pairs (Lut621/Chl *d*603 and Lut620/Chl *d*612) derived from the crystallographic structure of the native complex starting from a donor ^3^Chl *d* polarization P_*x*_:P_*y*_:P_*z*_ = 0.36:0.45:0.19 (see supporting Figure S4 and Table 2 for details). The TR-EPR spectra can be reconstituted with simulated components for the different triplets that contributed to the spectrum (Figure 2a-b and Table 2). A small amount of ^3^Chl *b* improved the spectral reconstitution, and its presence is congruent with the ODMR data (*vide infra*). A satisfactory concordance with the experimental spectra was achieved, with the corresponding relative contributions of ^3^Car and ^3^Chls *b* and *d* documented in Table 2. The presence of ^3^Chl is frequently identified in isolated native light-harvesting proteins, as well as in reconstituted complexes, particularly at low temperatures.^19,20,31,35−37^ However, it is also observed at physiological temperatures.^20,38^ In this study, we observe a slight increase in the quantity of unquenched ^3^Chls in the mutant compared to the WT, in contrast to observations in Lhcb-*ab*.^16^ It is important to highlight that the relative contribution documented in Table 2 does not reflect the populations of ^3^Car and ^3^Chl, due to the intrinsic different spin polarization of the spectra of Car and Chl.

**Table 2:** Simulation Parameters of the TR-EPR Spectra of Lhcb-*db* Variants in Figure 2 and of Chls *b* and d in Supporting Figure S4

		*D* [mT]	*E* [mT]	(*P*_*x*_:*P*_*y*_:*P*_*z*_)	(*L*_*x*_:*L*_*y*_:*L*_*z*_) [mT]	Percentage contribution to the absolute area
Lhcb-*db* WT	^3^Car (L1)	–41.4 ± 0.1	–3.90 ± 0.02	(0.42:0.20:0.38)	(2.5:2.5:2.5)	24
	^3^Chl *d*	27.3 ± 0.1	–5.35 ± 0.02	(0.36:0.45:0.19)	(3.0:3.0:3.0)	66
	^3^Chl *b*	31.4 ± 0.1	–4.28 ± 0.02	(0.34:0.42:0.24)	(3.5:3.5:3.5)	10
Lhcb-*db* A2	^3^Car (L2)	–40.7 ± 0.1	–4.20 ± 0.02	(0.42:0.21:0.37)	(2.5:2.5:2.5)	19
	^3^Chl *d*	27.3 ± 0.1	–5.35 ± 0.02	(0.36:0.45:0.19)	(3.0:3.0:3.0)	72
	^3^Chl *b*	31.4 ± 0.1	–4.28 ± 0.02	(0.34:0.42:0.24)	(3.5:3.5:3.5)	9
^3^Chl *d* in Tx-100 micelles		27.1 ± 0.1	–5.10 ± 0.02	(0.36:0.45:0.19)	(2.6:2.8:3.5)	
^3^Chl *b* in Tx-100 micelles		33.0 ± 0.1	–4.00 ± 0.02	(0.34:0.42:0.24)	(4.4:5.0:3.6)	

The mutation of the ligand of Chl 612 influences the line shape of the EPR spectra, owing to a change in the relative Chl/Car contribution and of the ^3^Car ZFS parameters, analogous to the case of antennae reconstituted with the natural complement of Chl *a/b* pigments.^16^ Significantly, the polarization pattern of ^3^Car remains unchanged and is the same observed in Lhcb-*ab*, suggesting that lutein is still capable of quenching the triplet states of Chls sitting at position 603. In order to acquire additional insights into the localization of triplet states within the reconstituted complexes, ODMR experiments were conducted. These experiments enable the identification of correlations between the magnetic and optical properties of the chromophores involved in triplet formation and probe the interactions among pigments in the vicinity of the molecules where the triplet states are localized.

As revealed by the TR- and pulse EPR spectra (Figure 2), the quenching of the Chl triplet state by the luteins is incomplete since unquenched ^3^Chl *d* is also detected. FDMR signals of LHCII-*db* WT and A2 have been detected by monitoring the fluorescence changes of the samples while sweeping the microwave frequency in the regions where the |D|-|E| and |D|+|E| transitions of ^3^Chl *d* states are expected (500–670 and 800–950 MHz, respectively, Figure 3a).^39,40^ WT and A2 display similar spectra, with |D|-|E| and |D|+|E| transitions peaking at 615 and 915 MHz, respectively. A very faint contribution at about 750 MHz is present in both WT and A2, and can be attributed to the |D|-|E| transition of a very small pool of unquenched ^3^Chl *b*.^41^

**Figure 3:**
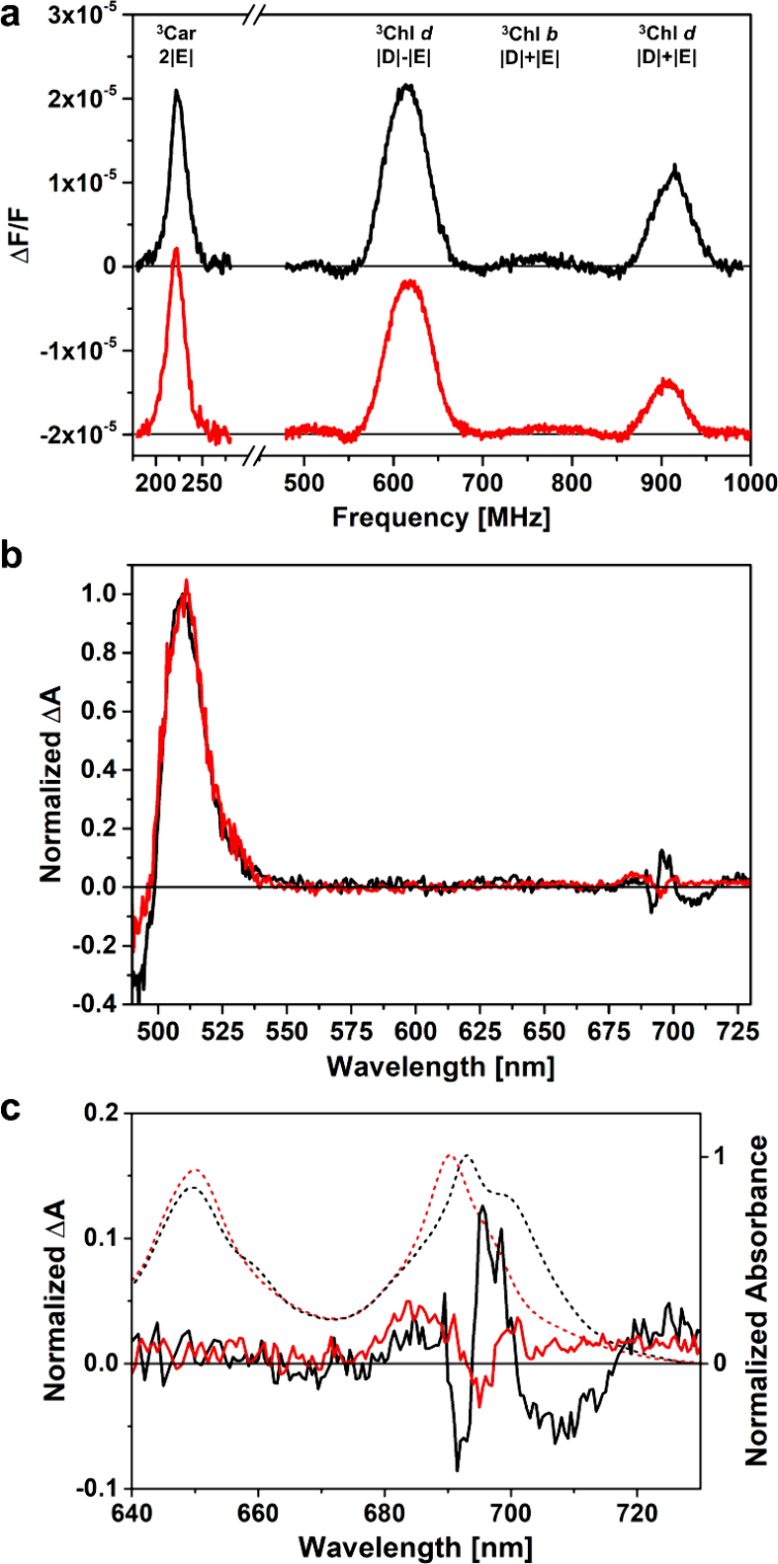
ODMR spectra at *T* = 1.8 K. (a) FDMR spectra of WT (black) and A2 (red) Lhcb-*db* recorded through a long-pass filter (λ > 680 nm). Amplitude modulation frequency 33 Hz in the 480–1000 MHz region, and 333 Hz in the 180–280 MHz region. Time constant 300 ms. (b) T-S spectrum of ^3^Car of WT (black) and A2 (red) Lhcb-*db*, obtained with a resonance frequency of 225 MHz (^3^Car 2|E| transition, see panel a). Amplitude modulation 333 Hz, time constant 1 s. (c) Enlarged *Q*_*y*_ Chl *b* and *d* absorption region, comparing the T-S (continuous line) and absorption (dashed line) spectra of WT (black) and A2 (red) Lhcb-*db*. The FDMR spectra are vertically shifted for better comparison, and the T-S and absorption spectra have been normalized for a better comparison.

^3^Car FDMR spectra have been performed in the frequency region where the intense 2|E| transition is expected (170–280 MHz).^42^ Carotenoids, which are nonfluorescent, are detected through changes of the Chl *d* emission, implying that an energy transfer processes connects the two pigments.^42^

The ^3^Car T-S spectra of the samples (Figure 3b) are obtained by fixing the microwave frequency at 225 MHz (maximum of the 2|E| transition in the ^3^Car FDMR spectra) while following the changes in the absorption. The spectra of the two samples show positive bands at about 510 nm, corresponding to the main band of the triplet–triplet absorption of Car species. In the same spectra, the structured bands that are present in the Chl Q_*y*_ absorption region were previously assigned to the interaction of the Car on which the triplet state is localized with nearby coupled Chls.^43−46^ This effect was recently explained by Migliore et al.^22^ showing that singlet-like triplet excitations can be described as effective singlet excitations on chlorophylls influenced by a stable electronic triplet on the carotenoid. When comparing these bands among the two samples, a different position of the bands is discernible (Figure 3c), with the disappearance in A2 of the redmost bleaching, attributed in Lhcb-*db* to an exciton state involving the Chl *d* 611–612–610 cluster. This is in agreement with the absorption spectrum (in Figure 3c, the 1.8 K absorption spectrum is reported in dashed lines for comparison). These results are congruent with a predominant contribution of the carotenoid in site L1 in the triplet quenching, and a substitution of it by L2 in the A2 mutant, where an excitonic interaction is still present between Chls *d* 603/602. The behavior is reminiscent of the analogous LHCII A2 variant reconstituted with Chls *a* and *b*.^16^

This investigation revealed that LHCII can sustain a remarkable alteration of its pigmentation, with the substitution of Chl *a* with Chl *d*, while preserving photoprotective TTET pathways. The same pigment pair (Chl 612/L1) retains the main photoprotective role, and the A2 mutation revealed that, analogously to the native complex, an alternative pigment pair (Chl 603/L2) is available whereas the main one is hindered in its triplet quenching activity. This further substantiates that LHCII possesses a resilient photoprotective mechanism,^17^ demonstrating sufficient flexibility to accommodate the substitution of various pigments while maintaining full functionality. Prior investigations have demonstrated that the replacement of luteins with alternative xanthophylls in the L1 and L2 sites of LHCII exhibits minimal to negligible impact on their capacity to execute their photoprotective function.^47,48^ In the case of the substitution of Chl *a* with Chl *d*, a small increase in the ^3^Chl/^3^Car intensity ratio can be observed while comparing the ^3^Car and ^3^Chl T-S spectra of LHCII-*db* with the corresponding spectra of LHCII-*ab*^16^ (see supporting Figure S6), indicating that the overall efficiency of the quenching is slightly less in LHCII-*db*, at least at the cryogenic temperatures investigated in this work. Since the TTET mechanism proceeds via a Dexter mechanism, it markedly depends on the overlap of the wave functions of the acceptor–donor pair.^15^ Therefore, slight structural rearrangements induced by the Chl *d* substitution, as well as alterations in the spin distribution of ^3^Chl *d* when compared to ^3^Chl *a* (that are expected, in light of the differences in their ZFS parameters^39,40^), could affect the TTET rate, and therefore the photoprotective efficiency. A previous molecular dynamic (MD) investigation of Chl *d* substituted LHCII revealed only minor reorientation of the bound Chls *d*,^11^ therefore a prevalent role of the differences in the spatial part of the ^3^Chl *d* wave function in causing the observed differences in the triplet quenching efficiencies is expected.

The flexibility of the complex’s photostability in terms of pigmentation is a pivotal requirement for the proposed utilization of LHCII and other LHCs in engineering plants capable of enhanced light harvesting in the far-red spectrum.^1−4^ Photoprotection is crucial for maintaining the structural and functional integrity of the photosynthetic apparatus. The demonstrated adaptability of LHCII to incorporate Chl *d* while maintaining its photoprotective optimized mechanisms provides a promising foundation for future efforts to expand the light-harvesting capabilities of plants into the far-red region of the spectrum.

## Materials and Methods

### Sample Preparation

Lhcb1 reconstitutions were performed following Natali et al.^49^ In short, Lhcb1 inclusion bodies were obtained from the *Escherichia coli* host strain BL21 transformed with the Lhcb1 construct containing the *Arabidopsis thaliana* Lhcb1.3 gene (AT1G29930), as previously described.^28^ LHCII A2 has an N183L mutation.

Chl *a* and *b*, and the native plant Cars were isolated from spinach. Chl *d* was extracted from *A. marina* cells as previously described.^50^ For the reconstitution with Chl *d*, the pigment mix was prepared by dissolving the pigments to obtain the desired composition of chlorophylls in 60 μL of ethanol per reconstitution (the Chl mix ratio was Chl *d*:*b* = 3:1), with a concentration of 8.3 mg/mL of Chl and 2.7 mg/mL of Cars. The reconstituted complexes were afterward purified by affinity chromatography and sucrose density gradient ultracentrifugation to remove unbound pigments.

Alongside the reconstitutions with Chl *d*, a reconstitution of Lhcb1 WT with Chl *a* was performed (with Chl ratio of *a*:*b* = 3:1). The reconstituted complex presented the same pigment composition as of the same complex in Remelli et al.^25^ as reported in Table 1.

For optical and electron magnetic resonance characterization, the samples were first concentrated in a discontinuous sucrose gradient and then with a 10 kDa Amicon Filter. The samples were shock-frozen in liquid nitrogen before being stored at −70 °C.

### Pigment Composition Analysis

Pigments were extracted from protein complexes using an 80%/20% acetone/water mixture. The Chl *b*/Chl *d* and Car/Chl ratios in the reconstituted samples were estimated by fitting the absorption spectrum of the pigment extract starting from the spectra of the isolated pigments in the same solvent, as reported in Elias et al.^11^ The Cars composition was analyzed by HPLC.

### Optical Spectroscopies

Room temperature absorption spectra were collected on a Varian Cary 4000 UV–Vis spectrophotometer at an OD of <1.0 cm^–1^. Room temperature fluorescence emission and excitation spectra were acquired on a HORIBA Jobin-Yvon Fluorolog-3 spectrofluorometer at an OD < 0.05 cm^–1^. Circular dichroism (CD) spectra were acquired on a Chirascan CD spectrophotometer at 10 °C at an OD of <1.0 cm^–1^. The sample concentration was adjusted to the required OD with a buffer containing 10 mM HEPES (pH 7.5), 0.5 M sucrose, and 0.06% βDM.

### TR- and Pulse EPR Experiments

X-band TR-EPR experiments were performed on a Bruker ELEXSYS E580 spectrometer, equipped with a dielectric cavity (Bruker ER 4117-DI5, TE_011_ mode), an Oxford CF935 liquid nitrogen flow cryostat, and an Oxford ITC4 temperature controller set at 80 K. The microwave frequency was measured by a frequency counter (HP5342A). The experiments were carried out in the absence of magnetic field modulation and using photoexcitation from a Nd:YAG pulsed laser (Quantel Brilliant) equipped with second- and third-harmonic modules (λ = 532 nm, pulse length = 5 ns, E/pulse 3.5 mJ, 10 Hz repetition time). The signal was recorded with a LeCroy 9300 digital oscilloscope, triggered by the laser pulse. For every field position, multiple transient signals were averaged. The spectra shown in this manuscript have been corrected for the off-resonance time response of the cavity and for the magnetic field baseline before the laser flash. The spectrometer used for pulse EPR experiments was the same for TR-EPR experiments, with an uncoupled resonator. Field-swept electron spin echo (FS-ESE) spectra were recorded using a 2p-ESE sequence (flash-DAF-π/2-τ–π–τ-echo) with a delay after the laser flash (DAF) of 300 ns. The length of the π/2 pulse was set to 16 ns and the delay τ was set to 300 ns. The FS-ESE spectra result from the integration of the full echo as a function of the magnetic field.

The LHCII samples were concentrated to ≈200 μg/mL of Chl. Glycerol, previously degassed by several cycles of freezing and pumping, was added (66% *v/v*) just before freezing to obtain a transparent matrix.

### Triplet State EPR Simulations

Simulations of the spin-polarized triplet spectra were performed using a program written in Matlab, with the aid of the EasySpin routine (6.0.0-dev.53),^51,52^ based on the full diagonalization of the triplet state spin Hamiltonian, including the Zeeman and electron–electron magnetic dipole interactions, considering a powder-like distribution of molecular orientations with respect to the magnetic field direction.^53^ The input parameters are the triplet state sublevel populations, the zero field splitting (ZFS) parameters, the line width, and the isotropic g value.

Calculations of the sublevel triplet state populations of the acceptor (Car), starting from those of the donor (Chl), were performed using a home-written program in Matlab software utilizing the atomic coordinates of LHCII (PDB-ID: 1RWT^26^). The program for the calculation of the triplet sublevel populations, previously described in great detail,^19,53^ adopts the limit of the high-field approximation and of a TTET process that is fast compared to the time evolution of the donor triplet spectrum (a process that requires hundreds of ns), but slow enough to allow spin alignment in the external magnetic field (a process that takes place on the picosecond time scale).

### ODMR Experiments

The principle of the ODMR technique has been previously reviewed in detail.^23,54^ Fluorescence- (FDMR) and absorption-detected (ADMR and triplet minus singlet, T-S) spectra were detected using a home-built setup, which was previously described.^55,56^ Briefly, the light of a tungsten lamp (250 W, Philips) was focused on the sample cell, which was immersed in a bath helium, after being filtered through either a 5 cm CuSO_4_ solution (FDMR spectra) or a 10 cm water filter (absorption-detected spectra). Fluorescence was detected by a photodiode in a 90° geometry with respect to the excitation light direction through a long-pass filter (λ > 680 nm), while in ADMR/T-S experiments the transmittance was detected in a standard straight geometry through a monochromator (Jobin Yvon HR250). The microwave resonator, where the sample cell is inserted, consists of a slow pitch helix. The microwaves (MW source HP8559b, sweep oscillator equipped with a HP83522s plug-in and amplified by a TWT Sco-Nucletudes mod 10–46–30 amplifier) were on/off amplitude modulated for selective amplification through a Lock-In amplifier (EG&G, mod 5210).

Glycerol was added (66% *v/v*) to the LHCII samples immediately before the insertion into the cryostat to avoid sample degradation (final concentration ≈100 μg/mL of Chl). In all measurements, the temperature was kept at 1.8 K.

## Supporting Information Available

Circular Dichroism spectra, fluorescence excitation spectra, complete TR-EPR surfaces, TR-EPR spectra of isolated Chl *b* and Chl *d* in detergent micelles, additional pulse EPR spectra, comparison of Lhcb-*db* and Lhcb-*ab* T-S spectra (PDF)
Transparent Peer Review report available (PDF)

## Abbreviations

Car: carotenoid
Chl: chlorophyll
FDMR: fluorescence detected magnetic resonance
FS-ESE: field-swept electron spin echo
Lut: lutein
LHCII: light-harvesting complex of photosystem II
Neo: neoxanthin
ODMR: optically detected magnetic resonance
T-S: triplet minus singlet
TR-EPR: time-resolved electron paramagnetic resonance
TTET: triplet–triplet energy transfer
Vio: violaxanthin
ZFS: zero-field splitting
