# Supplementary material for: Far-Red Absorbing LHCII Incorporating Chlorophyll d Preserves Photoprotective Carotenoid Triplet–Triplet Energy Transfer Pathways
Source: J Phys Chem Lett. 2025 Feb 10;16(7):1720–8. doi: 10.1021/acs.jpclett.4c03463 (PMC11849036; doi:10.1021/acs.jpclett.4c03463)
Supplement: Supplementary file 1 — jz4c03463_si_001.pdf [file jz4c03463_si_001.pdf]

SUPPORTING INFORMATION

Far-red absorbing LHCII incorporating chlorophyll *d* preserves photoprotective carotenoid triplet-triplet energy transfer pathways

Niccolò Cianfarani[a,b,§], Andrea Calcinoni[a,§], Alessandro Agostini[a,*], Eduard Elias[b], Marco Bortolus[a], Roberta Croce[b], Donatella Carbonera[a,*]

[a]Department of Chemical Sciences, University of Padova, via Marzolo 1, 35131 Padova, Italy

[b]Biophysics of Photosynthesis, Department of Physics and Astronomy, Faculty of Science, Vrije Universiteit Amsterdam and LaserLaB Amsterdam, De Boelelaan 1100, 1081 HZ Amsterdam, the Netherlands

[§]Equally contributed to this work

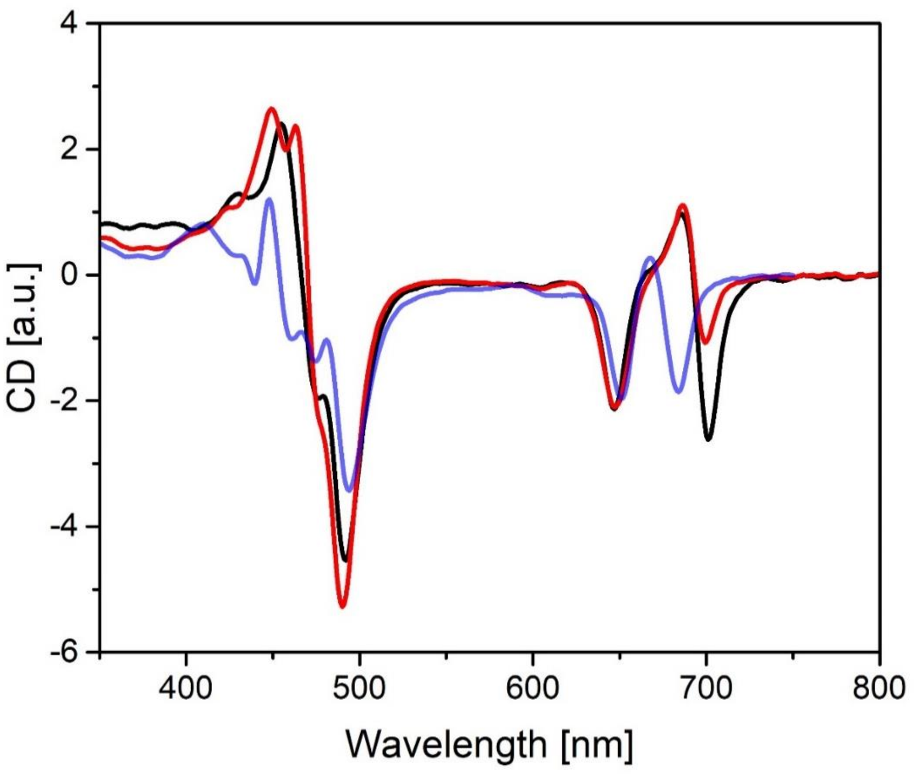

Supplementary Figure S1. Circular Dichroism spectra. Lhcb-*db* WT (black line), Lhcb-*db* A2 (red line), and Lhcb-*ab* (blue line) collected at 283K. The spectra are scaled to the normalized Q_y peaks of the corresponding absorption spectra.

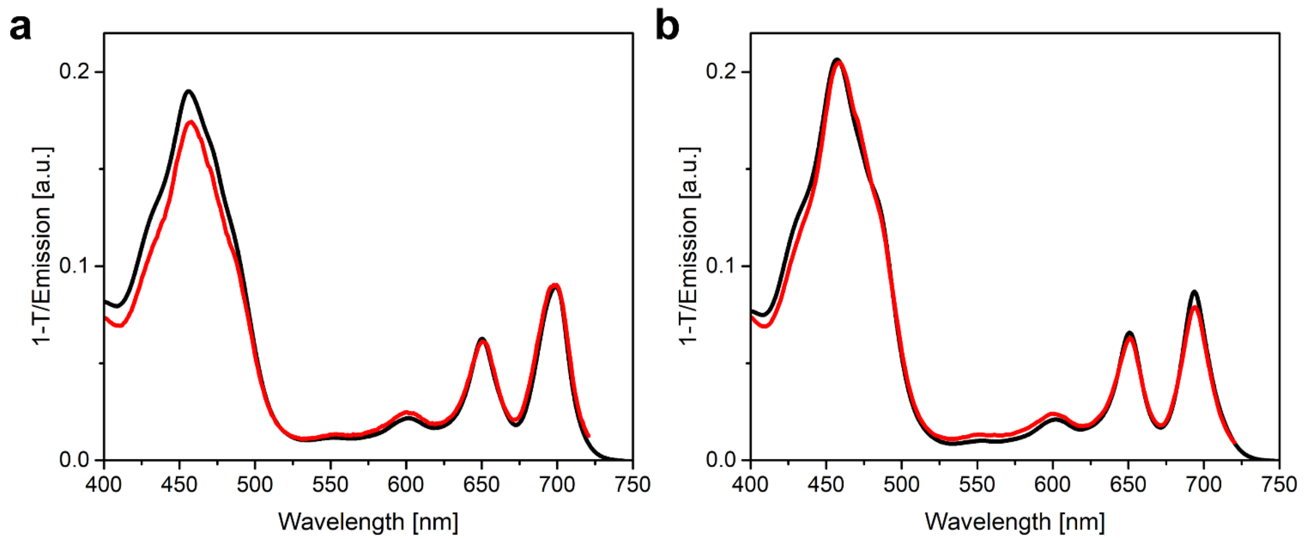

Supplementary Figure S2. 1-T (Transmittance) vs fluorescence excitation spectra (black and red lines, respectively). LHCII-*db* WT **(a)** and A2 **(b)** measured with emission detected at 760 nm.

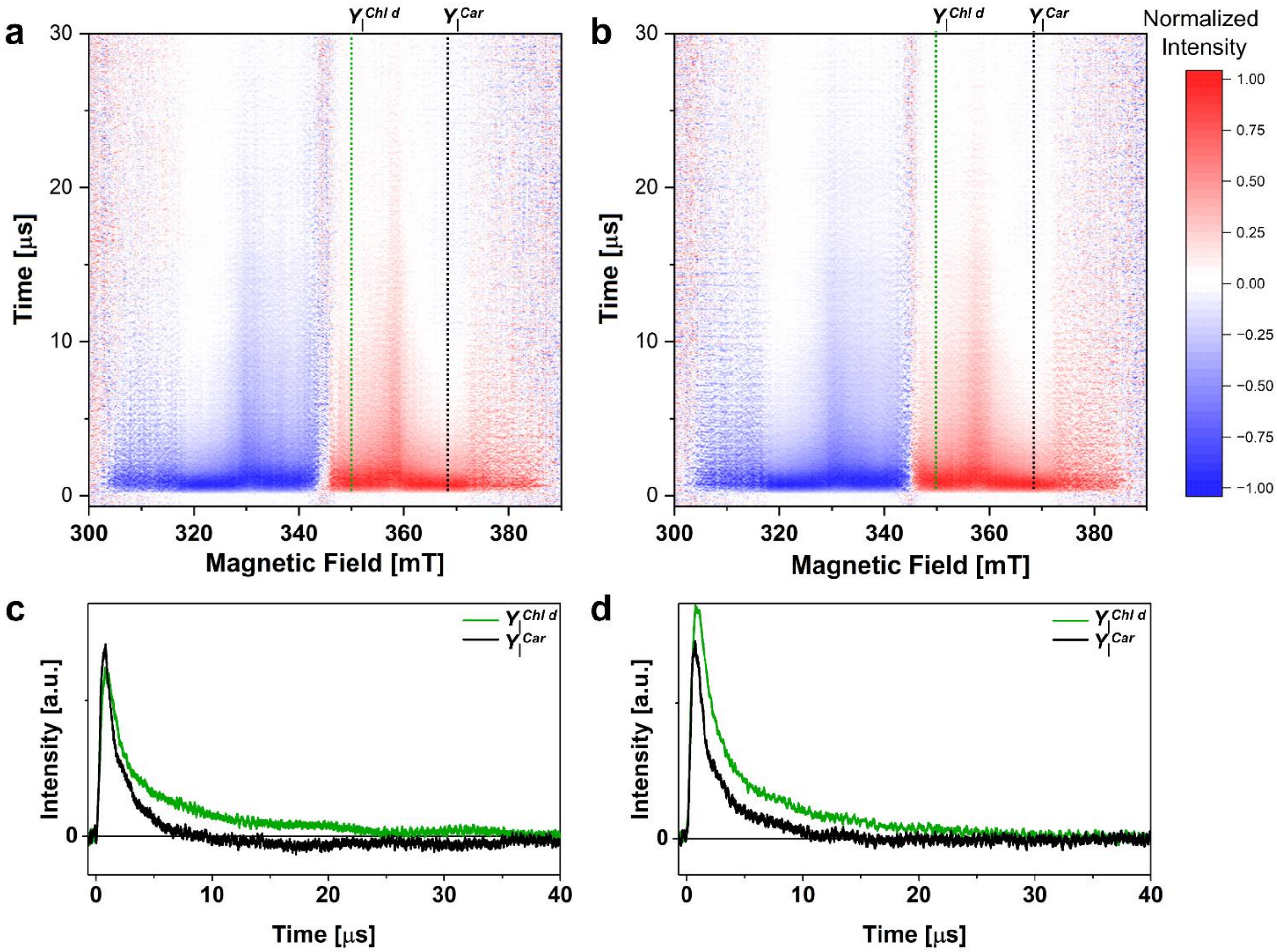

Supplementary Figure S3. X-band TR-EPR surfaces of Lhcb-*db* WT **(a)** and A2 **(b)** in glycerol-water buffer (66 % v/v) glass at 80 K. The transients corresponding to the $Y_{|}^{Chl\ d}$ (green line) and $Y_{|}^{Car}$ (black line) transitions are plotted below (Lhcb-*db* WT and A2 in panels **c** and **d**, respectively).

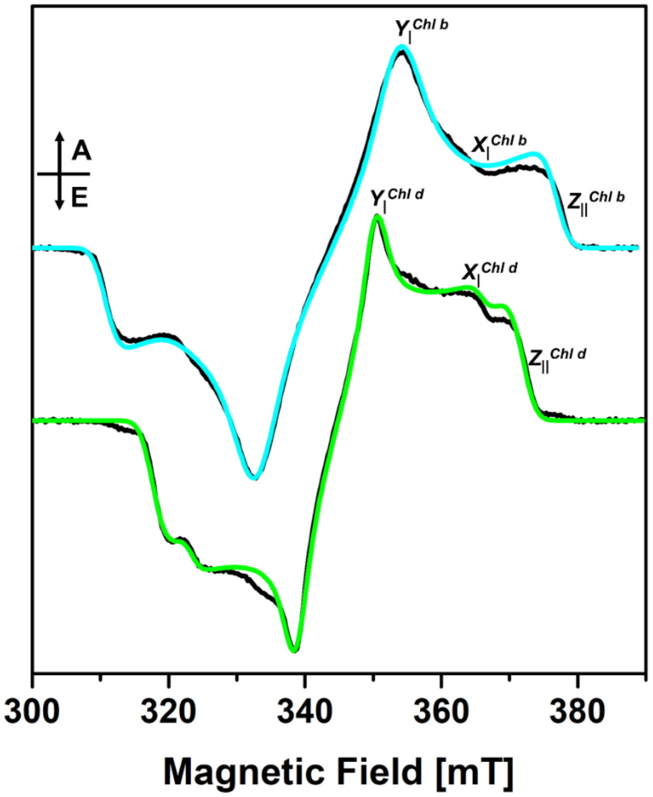

Supplementary Figure S4. X-band TR-EPR spectra of Chl *b* and Chl *d* in Tx-100 micelles (black lines), taken 700 ns after the laser pulse. The spectra have been collected at 80 K. *X*, *Y*, and *Z* represent the ZFS canonical orientations; the high field transitions are labeled. The simulated triplet spectra (cyan and green lines for Chl *b* and *d*, respectively) were calculated using the parameters reported in Table 2.

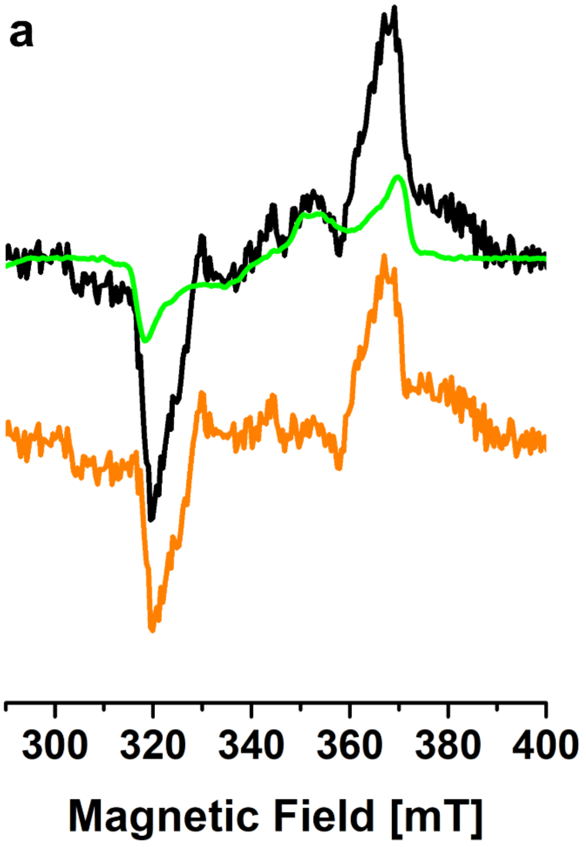

Supplementary Figure S5. X-band pulse EPR spectra. FS-ESE spectra (black lines) of LHCII-*db* WT **(a)** and A2 **(b)** and of Chl *d* dissolved in Triton X-100 micelles (green lines) at 80 K. The differences (orange lines) between the FS-ESE spectra, which corresponds to the 'pure' ^{3}Car spectrum, has been vertically translated for clarity.

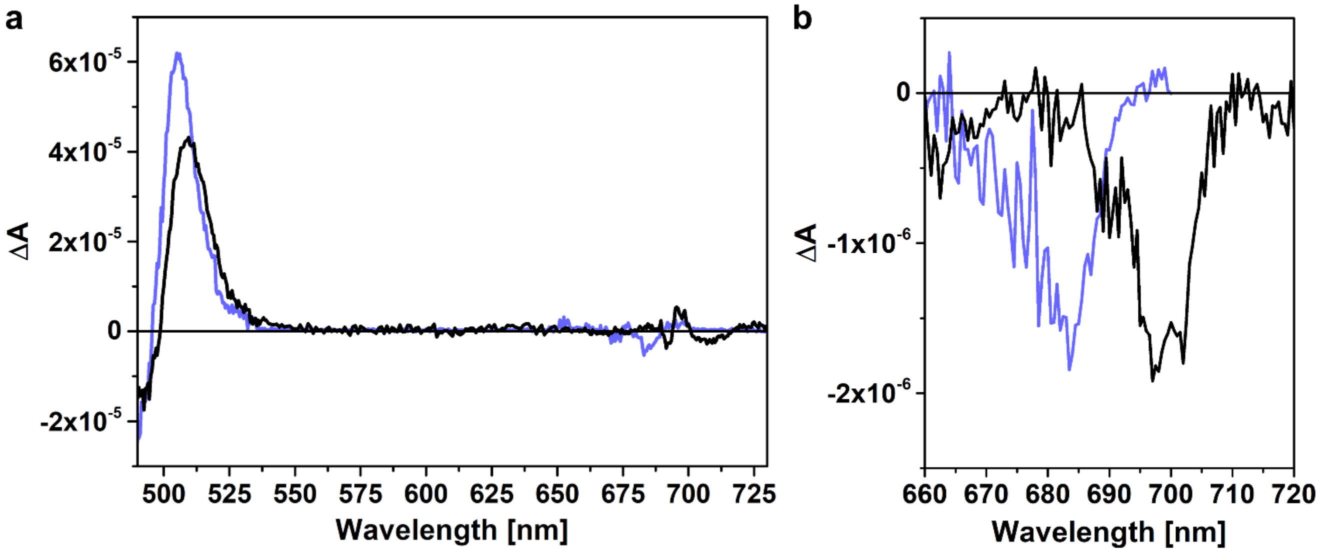

Supplementary Figure S6. Comparison of T-S spectra. (a) ^{3}Car T-S spectra of Lhcb-*db* WT (black line) and Lhcb-*ab*[1] WT (blue line), obtained with a resonance frequency of 225 MHz (^{3}Car 2|E| transition, see figure 3a). Amplitude modulation frequency 333 Hz, time constant 1 s, temperature 1.8 K. in the 480-1000 MHz region, and 333Hz in the 180-280 MHz region. **(b)** ^{3}Chl T-S spectra of Lhcb-*db* WT (black line) and Lhcb-*ab*[1] WT (blue line), obtained with a resonance frequency of either 615 or 740[1] MHz (^{3}Chl *d* and ^{3}Chl *a* |D|-|E| transition, respectively). Amplitude modulation frequency 33 Hz, time constant 300 ms, temperature 1.8 K.

References

(1) Agostini, A.; Nicol, L.; Da Roit, N.; Bortolus, M.; Croce, R.; Carbonera, D. Altering the Exciton Landscape by Removal of Specific Chlorophylls in Monomeric LHCII Provides Information on the Sites of Triplet Formation and Quenching by Means of ODMR and EPR Spectroscopies. *Biochim. Biophys. Acta - Bioenerg.* **2021**, *1862* (11), 148481. https://doi.org/10.1016/j.bbabio.2021.148481.
