# Supplementary material for: Far-Red Absorbing LHCII Incorporating Chlorophyll d Preserves Photoprotective Carotenoid Triplet–Triplet Energy Transfer Pathways
Source: J Phys Chem Lett. 2025 Feb 10;16(7):1720–8. doi: 10.1021/acs.jpclett.4c03463 (PMC11849036; doi:10.1021/acs.jpclett.4c03463)
Supplement: Supplementary file 2 — jz4c03463_si_002.pdf [file jz4c03463_si_002.pdf]

jz-2024-03463j

Name: Peer Review Information for "Far-red absorbing LHCII incorporating chlorophyll *d* preserves photoprotective carotenoid triplet-triplet energy transfer pathways"

First Round of Reviewer Comments

Reviewer: 1

Comments to the Author
The manuscript describes the characterization of LHCII photosynthetic antenna complexes in which the native chlorophyll a has been replaced with chlorophyll d. Using time-resolved EPR and optically detected magnetic resonance methods the authors show convincingly that triplet-triplet energy transfer from Chl d to the carotenoids takes place. Thus, demonstrating that the photoprotection mechanisms are not affected by the change in chlorophyll.
This is important for the proposed bioengineering of red-light absorbing plants.

There are a couple of discussion points the authors should address.

1. Some discussion of the factors that affect the rate of triplet-triplet energy transfer is needed and an explanation of whether any these might be expected to be different with the change of chlorophyll.

2. All of the data have been collected at cryogenic temperatures which are far from native conditions. Could the observation that not all 3Chl is quenched by energy transfer to the carotenoids be a result of the low temperature? How well can the low temperature results be correlated to actual biological conditions?

A couple of very minor points

Page 6 line 44 and SI Figure S2. I think the notation 1-T may not be familiar to all readers and it would be useful to explain that T = transmittance.

The notation “A2” mutant should be explained for non-experts. i.e. The notation A1, A1, etc for the binding sites and the proper amino acid notation for the point mutation should be given with an explanation of why it is referred to as “A2”.

Reviewer: 2

Comments to the Author
Cianfarani et al. investigated the incorporation of chlorophyll d (chl-d) into the light-harvesting complex II monomers (Lhcb1), aiming to extend the spectral range of the major

light-harvesting pigment-protein complex to far-red wavelengths. Steady-state and time-resolved EPR (TR-EPR) and optically detected paramagnetic resonance (ODMR) spectroscopies were used to explore the chl-d incorporation. The key aim of this study was to explore whether incorporation of Chl-d still facilitated chlorophyll-to-carotenoid triplet energy transfer (TTET) to mitigate harmful oxygen sensitization processes leading to photodamage. The authors claim that this process is just as efficient in the chl-d protein as the chl-a containing (native) protein.

However, the authors fail to quantify the extent of the TTET, and thus the efficiency of the important process, and therefore this claim is not founded:

(i) TR-EPR and ODMR experiments show that TTET from chlorophyll to carotenoids occurs- this is without doubt. But as presented, only a qualitative measure is given, rather than quantitative, e.g. the authors are unable to determine the rate of TTET in the chl-d containing WT or A2 Lhcb1 complexes, as they monitor a fixed point in time of the kinetics (due to the inherent limit on TR-EPR time resolution) and are unable to follow the whole decay, nor extract a quantum yield for the process. Therefore, it is unclear whether this part of the energy landscape is truly similar to the chl-a containing natural complex, or as claimed 'efficient'. Therefore, the study as presented is too speculative.
(ii) There are assumptions made throughout that the physical structure of the chl-d containing Lhcb1 complex is the same as the native (chl-a containing) form, but no crystal structure is given to prove this is correct. This is critical when evaluating the A2 mutant when compared to WT.
(iii) Whether or not this is an important endeavor is an important question- the authors imply that light harvesting is one of the limiting factors in plants. However, it is well established, quoting from Ort et al.: "A principal limitation of efficient photosynthesis is that organisms absorb more light in full sunlight than they can use productively." https://doi.org/10.1073/pnas.1424031112

For the reasons outlined above, this paper is a worthy contribution to the scientific literature, but is only to a selective audience, and therefore more appropriate in a specialized scientific journal.

Author's Response to Peer Review Comments:

The authors thank the reviewers for their detailed comments that helped us improve the clarity of the presentation in the revised version of the manuscript. We have addressed all points in detail as listed below and have accordingly modified the manuscript, adding two figures in the supporting information. Reviewers' comments in this response letter are printed in black and our responses in blue. All modifications are also visible in a marked-up version of the manuscript. We hope that with our responses and modifications, the manuscript can now be recommended for publication.

Reviewer(s)' Comments to Author:

Reviewer: 1

Recommendation: This paper is publishable subject to minor revisions noted. Further review is not needed.

Comments:
The manuscript describes the characterization of LHCII photosynthetic antenna complexes in which the native chlorophyll a has been replaced with chlorophyll d. Using time-resolved EPR and optically detected magnetic resonance methods the authors show convincingly that triplet-triplet energy transfer from Chl d to the carotenoids takes place. Thus, demonstrating that the photoprotection mechanisms are not affected by the change in chlorophyll. This is important for the proposed bioengineering of red-light absorbing plants.

There are a couple of discussion points the authors should address.

1. Some discussion of the factors that affect the rate of triplet-triplet energy transfer is needed and an explanation of whether any these might be expected to be different with the change of chlorophyll.

Following the reviewer's suggestion, we addressed this point at page 13:

"Since the TTET mechanism proceeds via a Dexter mechanism, it markedly depends on the overlap of the wavefunctions of the acceptor-donor pair.[15] Therefore, slight structural rearrangements induced by the Chl d substitution, as well as alterations in the spin distribution of ^{3}Chl d when compared to ^{3}Chl a (that are expected, in light of the differences in their ZFS parameters), could affect the TTET rate, and therefore the photoprotective efficiency. To analyze in more details these differences, the determination of the structure of LHCs of altered pigmentation would pave the way to ulterior computational and spectroscopic investigations."

2. All of the data have been collected at cryogenic temperatures which are far from native conditions. Could the observation that not all 3Chl is quenched by energy transfer to the carotenoids be a result of the low temperature? How well can the low temperature results be correlated to actual biological conditions?

The reviewer is right. The low temperatures at which the measurements have been carried out are expected to reduce the triplet quenching efficiency, increasing the unquenched ^{3}Chl yield. In LHCII containing Chls *a* and *b*, the triplet quenching efficiency was found to be 95% at room temperature, leaving about 5% of the chlorophyll triplets unquenched (Mozzo *et al.* 2008, doi:10.1074/jbc.M708961200), whereas it is reduced to 82% at 4 K, with an increasing amount of unquenched ^{3}Chls being detected (Peterman *et al.* 1995, doi: 10.1016/S0006-3495(95)80138-4). Therefore, the observation of unquenched ^{3}Chls, enhanced by the cryogenic temperature likely because the activated energy transfer steps among pigments are less efficient, is a common finding for LHCs, even at room temperature (Mozzo *et al.* 2008; Kvíčalová et al. 2016, doi:10.1016/j.bbabio.2016.01.008). This line of discussion has been integrated in the manuscript at page 9, as follows:

"The presence of ^{3}Chl is commonly observed in isolated native light-harvesting proteins as well as in reconstituted complexes, especially at low temperature[16,21,31,35–37] but at physiological temperature as well.[21,38]"

A couple of very minor points
Page 6 line 44 and SI Figure S2. I think the notation 1-T may not be familiar to all readers and it would be useful to explain that T = transmittance.

Done

The notation "A2" mutant should be explained for non-experts. i.e. The notation A1, A1, etc for the binding sites and the proper amino acid notation for the point mutation should be given with an explanation of why it is referred to as "A2".

We liked the suggestion of the reviewer to explain the origin of the name of the mutant to the readers and to introduce the nature of the point mutation outside of the M&M section. Therefore, we have reworded as follows the sentence in which the mutant is first introduced:

"Alongside LHCII WT, we also characterize the A2 mutant in which the Chl 612 binding site is removed[25] *by means of a N183L mutation directed at preventing its magnesium-ligation (we will refer to the Chl-binding sites following Liu et al.*[26] *throughout the manuscript. Note that the 612 binding site corresponds to the A2 site in Khulbrandt et al.*[27]*)."*

That said, we would prefer to stick to a single notation for the other Chl-binding positions mentioned in the manuscript, and we think that panels **a** and **b** of **Figure 1** are sufficient to clarify the nomenclature adopted through the manuscript, that is the one introduced by Liu *et al*.

Urgency: Top 10% Significance: Top 10% Novelty: Top 10% Scholarly Presentation: Top 10%

Is the paper likely to interest a substantial number of physical chemists, not just specialists working in the authors' area of research?: Yes

Reviewer: 2

Recommendation: Reconsider as an article in The Journal of Physical Chemistry A/B/C.

Comments:
Cianfarani et al. investigated the incorporation of chlorophyll d (chl-d) into the light-harvesting complex II monomers (Lhcb1), aiming to extend the spectral range of the major light-harvesting pigment-protein complex to far-red wavelengths. Steady-state and time-resolved EPR (TR-EPR) and optically detected paramagnetic resonance (ODMR) spectroscopies were used to explore the chl-d incorporation. The key aim of this study was to explore whether incorporation of Chl-d still facilitated chlorophyll-to-carotenoid triplet energy transfer (TTET) to mitigate harmful oxygen sensitization processes leading to photodamage. The authors claim that this process is just as efficient in the chl-d protein as the chl-a containing (native) protein.

However, the authors fail to quantify the extent of the TTET, and thus the efficiency of the important process, and therefore this claim is not founded:

(i) TR-EPR and ODMR experiments show that TTET from chlorophyll to carotenoids occurs- this is without doubt. But as presented, only a qualitative measure is given, rather than quantitative, e.g. the authors are unable to determine the rate of TTET in the chl-d containing WT or A2 Lhcb1 complexes, as they monitor a fixed point in time of the kinetics (due to the inherent limit on TR-EPR time resolution) and are unable to follow the whole decay, nor extract a quantum yield for the process. Therefore, it is unclear whether this part of the energy landscape is truly similar to the chl-a containing natural complex, or as claimed 'efficient'. Therefore, the study as presented is too speculative.

A precise determination of the rate of TTET in the complexes of interest would require a different experimental setup, however from the analysis of the LHCII-*db* ^{3}Car TR-EPR spectrum it is clear that the transfer occurs within the time resolution of the instrumental response as in the case of LHCII-*ab,* since no correlation was found between the disappearance of the ^{3}Chl and the rising of the ^{3}Car signals (see panels **c** and **d** of the following figure). Prompted by this question of the reviewer, we decided to include in the supplementary

material section of the manuscript the complete time evolution of the TR-EPR signal, as well as the two most diagnostic transient spectra in panels **c** and **d**. This is now the supplementary figure S3.

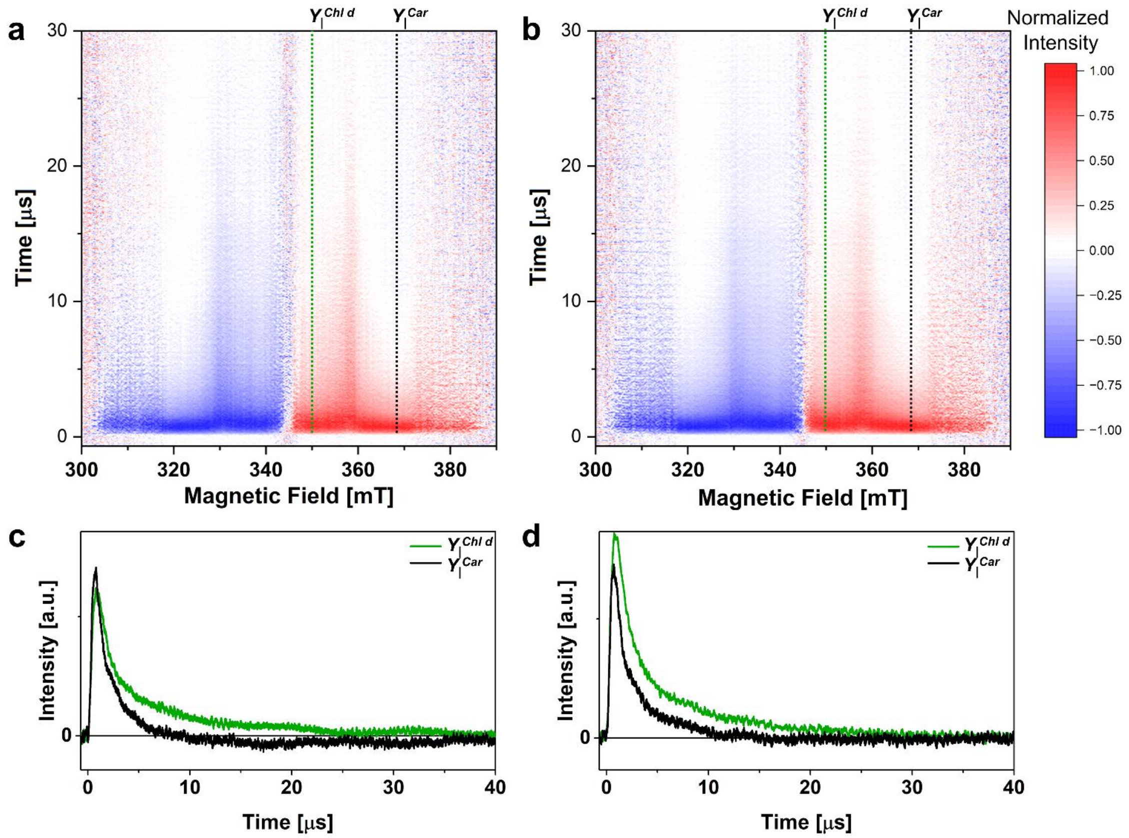

Supplementary Figure S3. X-band TR-EPR surfaces of Lhcb-*db* WT **(a)** and A2 **(b)** in glycerol-water buffer (66 % v/v) glass at 80 K. The transients corresponding to the $Y_{|}^{Chl\ d}$ (green line) and $Y_{|}^{Car}$ (black line) transitions are plotted below (Lhcb-*db* WT and A2 in panels **c** and **d**, respectively).

Regarding the part of the question dealing with the actual relative amount of ^{3}Car and its comparison with the amount of unquenched ^{3}Chls, Absorption Detected Magnetic Resonance can provide a valuable measure. From the comparison of the ^{3}Car and ^{3}Chl T-S spectra collected on the Chl *d*-containing Lhcb1 with the corresponding spectra detected on the Chl *a*-containing complex, a similarity in the ^{3}Car/^{3}Chl ratio can be extracted. We added the following figure to the Supporting Information section of the manuscript, in which the signals are shown without normalization, and at page 13 we added the following paragraph, addressing this point:

"In the case of the substitution of Chl a with Chl d, a small increase in the ^{3}Chl/^{3}Car intensity ratio can be observed while comparing the ^{3}Car and ^{3}Chl T-S spectra of LHCII-db with the corresponding spectra of LHCII-ab[16] (see supporting Figure S5), indicating that the overall efficiency of the quenching is slightly less in LHCII-db, at least at the cryogenic temperatures investigated in this work. Since the TTET mechanism proceeds via a Dexter mechanism, it markedly depends on the overlap of the wavefunctions of the acceptor-donor pair.[15] Therefore, slight structural rearrangements induced by the Chl d substitution, as well as alterations in the spin distribution of ^{3}Chl d when compared to ^{3}Chl a (that are expected, in light of the differences in their ZFS parameters[39,40]), could affect the TTET rate, and therefore the photoprotective efficiency. Since molecular dynamic (MD) investigations of Chl d substituted LHCII revealed only minor reorientation of the bound Chls d,[11] significant structural rearrangements can be ruled out, suggesting that the observed differences in the triplet quenching efficiencies are to be assigned to differences in the spatial part of the ^{3}Chl d wavefunction."

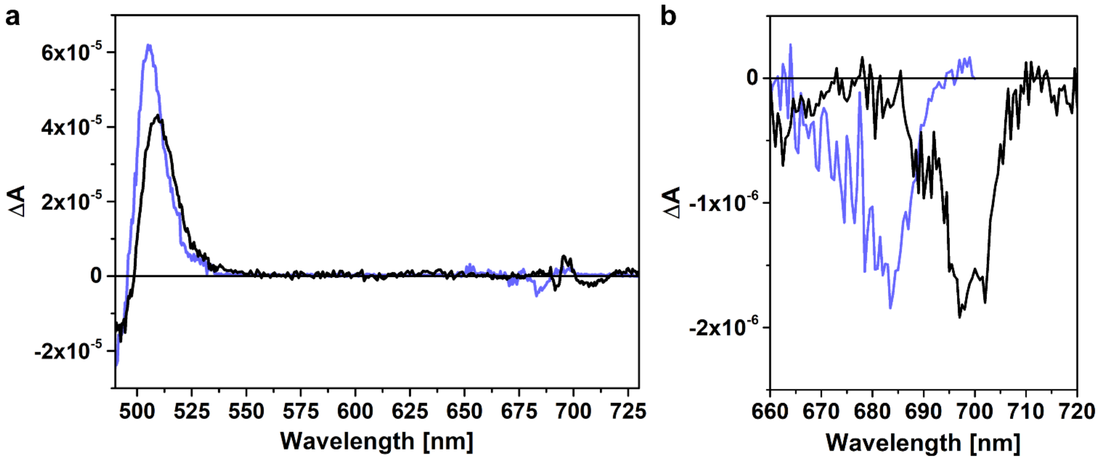

Supplementary Figure S6. Comparison of T-S spectra. (a) ^{3}Car T-S spectra of Lhcb-*db* WT (black line) and Lhcb-*ab*^{2} WT (blue line), obtained with a resonance frequency of 225 MHz (^{3}Car 2|E| transition, see figure 3a). Amplitude modulation frequency 333 Hz, time constant 1 s, temperature 1.8 K. in the 480-1000 MHz region, and 333Hz in the 180-280 MHz region. **(b)** ^{3}Chl T-S spectra of Lhcb-*db* WT (black line) and Lhcb-*ab*^{2} WT (blue line), obtained with a resonance frequency of either 615 or 740^2 MHz (^{3}Chl *d* and ^{3}Chl *a* |D|-|E| transition, respectively). Amplitude modulation frequency 33 Hz, time constant 300 ms, temperature 1.8 K.

(ii) There are assumptions made throughout that the physical structure of the chl-d containing Lhcb1 complex is the same as the native (chl-a containing) form, but no crystal structure is given to prove this is correct. This is critical when evaluating the A2 mutant when compared to WT.

In the manuscript in which the Chl *d*-reconstituted LHCII complexes were originally presented, a thorough molecular dynamic investigation has been carried out (Elias et al. 2021, doi:10.1021/acs.biomac.1c00435). In there, it has been shown that there are no important conformational differences upon the substitution of Chl *a* with Chl *d,* and that the coupling between pigments remains the same, showing that the LHCII architecture is conserved. This point is now addressed at page 13:

"Since molecular dynamic (MD) investigations of Chl d substituted LHCII revealed only minor reorientation of the bound Chls d,[11] *significant structural rearrangements can be ruled out"*

The EPR characterization carried out in our work strengthens this previous finding, since the polarization of the ^{3}Car pulse EPR spectra provides a strong indication of a conserved relative geometry of the Chl-Car donor-acceptor pairs at the heart of the complex. Since this is the portion of the pigment-protein complex on which the present spectroscopic investigation is focused on, utilizing the LHCII *ab* complex structure as a structural model is confirmed to be a reasonable assumption. Moreover, we would like to remark that in recent years there has been a growing number of structures of LHCs characterized by different pigments in terms of their bound Chls: FCPs with Chls *c* occupying sites 603 and 612 (Wang *et al.* 2019, doi: 10.1126/science.aav0365), and more recently SCP in which Chl *b* occupies several sites that are occupied by Chl *a* in LHCII (Seki *et al.* 2022, doi:10.1016/j.bbadva.2022.100064). Inspecting their structures, a remarkable conservation of the overall disposition of the pigment orientations is observed, particularly the two clusters mostly discussed in the present paper (i.e., Chls 602-603-609 and 610-612-611).

(iii) Whether or not this is an important endeavor is an important question- the authors imply that light harvesting is one of the limiting factors in plants. However, it is well established, quoting from Ort et al.: "A principal limitation of efficient photosynthesis is that organisms absorb more light in full sunlight than they can use productively." https://doi.org/10.1073/pnas.1424031112

There are several possible optimizations when it comes with the efficient harvest and utilization of light. While in full sunlight, light-harvesting is not the limiting factor, there are many conditions in which it is, such as

under the canopy, where only far-red light is available. In those conditions far-red absorption is an advantageous trait, as can be observed by the natural adaptations of plants (Li et al. 2024, doi:10.1038/s41467-024-50655-9), algae (Koehne et al. 1999, doi: 10.1016/S0005-2728(99)00061-4; Kotabová et al. 2014, doi:10.1016/j.bbabio.2014.01.012; Wolf et al. 2018, doi:10.1007/s11120-017-0401-z), and cyanobacteria (Miyashita et al. 1996, doi: 10.1038/383402a0; Behrendt et al. 2015, doi:10.1038/ismej.2015.14) living in ecological niches characterized by light spectra enriched in the far-red wavelengths. Indeed Ort et al. have also a part of the perspective dedicated to the creation of a "smart canopy", i.e. crops characterized by an increased far-red light absorption in the lower part of the canopy, which is considered one of the options to increase crop productivity via the optimization of photosynthesis (Ort et al. 2015, doi: 10.1073/pnas.1424031112; and Croce et al. 2024, 10.1093/plcell/koae132).

We modified the introduction in order to better highlight this important message:

"It has been estimated that the introduction of far-red-absorbing chlorophylls (either Chl d or Chl f) into LHCII could allow to achieve a 19% gain in photosynthetic efficiency.[1] *Previous studies have found that Chl d can be successfully introduced in reconstituted LHCII, with minimal interference with the excited state decay or energy equilibration processes within the complex, paving the way for engineering plant LHCs capable of enhanced light harvesting in the far-red,*[11] *with the goal of achieving a "smart canopy"*[3]*, i.e. crops characterized by an increased far-red light absorption in the lower part of the canopy."*

For the reasons outlined above, this paper is a worthy contribution to the scientific literature, but is only to a selective audience, and therefore more appropriate in a specialized scientific journal.

Urgency: Low Significance: Moderate Novelty: Moderate Scholarly Presentation: Moderate

Is the paper likely to interest a substantial number of physical chemists, not just specialists working in the authors' area of research?: No

Second Round of Reviewer Comments

Reviewer: 2

Comments to the Author
The authors have revised the manuscript, changing the 'pitch' of the paper to address my comment that light harvesting is not the limiting step under the regular solar spectrum.

Secondly, some quantification of the relative triplet carotenoid yield is provided through analysis of the T-S spectrum in the SI. This is a difficult to fully quantify due to the limited time resolution associated with the technique, however. The associated additional text in the manuscript is not as clear as it could be:

"Since the TTET mechanism proceeds via a Dexter mechanism, it markedly depends on the overlap of the wavefunctions of the acceptor-donor pair.15 Therefore, slight structural rearrangements induced by the Chl d substitution, as well as alterations in the spin distribution of 3Chl d when compared to 3Chl a (that are expected, in light of the differences in their ZFS parameters39,40), could affect the TTET rate, and therefore the photoprotective efficiency."

The authors recognise there are differences between the two samples and thus the yields vary. These could originate in altered binding configurations for chl-d. Then the authors introduce MD results:

"Since molecular dynamic (MD) investigations of Chl d substituted LHCII revealed only minor reorientation of the bound Chls d,11 significant structural rearrangements can be ruled out, suggesting that the observed differences in the triplet quenching efficiencies are to be assigned to differences in the spatial part of the 3Chl d wavefunction."

But what is the accuracy of the MD forcefield for chl-d relative to chl-a? A simpler argument is surely there are small structural changes, MD agrees with this, and this results in a change in the spatial part of the wavefunction, explaining all the experimental observations. Can the authors please condense this paragraph to a single and simple argument, such as the one I have outlined, to improve readability.

Author's Response to Peer Review Comments:

Reviewer(s)' Comments to Author:

Reviewer: 2

Recommendation: This paper is publishable subject to minor revisions noted. Further review is not needed.

Comments:
The authors have revised the manuscript, changing the 'pitch' of the paper to address my comment that light harvesting is not the limiting step under the regular solar spectrum.

Secondly, some quantification of the relative triplet carotenoid yield is provided through analysis of the T-S spectrum in the SI. This is a difficult to fully quantify due to the limited time resolution associated with the technique, however. The associated additional text in the manuscript is not as clear as it could be:
"Since the TTET mechanism proceeds via a Dexter mechanism, it markedly depends on the overlap of the wavefunctions of the acceptor-donor pair.[15] Therefore, slight structural rearrangements induced by the Chl d substitution, as well as alterations in the spin distribution of ^{3}Chl *d* when compared to ^{3}Chl *a* (that are expected, in light of the differences in their ZFS parameters[39,40]), could affect the TTET rate, and therefore the photoprotective efficiency."

The authors recognise there are differences between the two samples and thus the yields vary. These could originate in altered binding configurations for chl-d. Then the authors introduce MD results:

"Since molecular dynamic (MD) investigations of Chl *d* substituted LHCII revealed only minor reorientation of the bound Chls *d*,[11] significant structural rearrangements can be ruled out, suggesting that the observed differences in the triplet quenching efficiencies are to be assigned to differences in the spatial part of the ^{3}Chl *d* wavefunction."

But what is the accuracy of the MD forcefield for chl-d relative to chl-a? A simpler argument is surely there are small structural changes, MD agrees with this, and this results in a change in the spatial part of the wavefunction, explaining all the experimental observations. Can the authors please condense this paragraph to a single and simple argument, such as the one I have outlined, to improve readability.

We disagree on this point with the reviewer, the structural changes found in the various MD trajectories are very small (see figure S5 in Elias *et al.* 2021) and the differences in the ^{3}Chl *d* triplet wavefunction distribution revealed by the differences in the ZFS are most likely the source of the difference between the Chl *a* and Chl *d* reconstituted LHCs. We reworded the sentence in order to highlight that the latter one is the prevalent contribution but that a minor one from subtle reorganizations could still play a role in the overall triplet quenching efficiency, as follows:

"Therefore, slight structural rearrangements induced by the Chl d substitution, as well as alterations in the spin distribution of ^{3}Chl d when compared to ^{3}Chl a (that are expected, in light of the differences in their ZFS parameters[39,40]), could affect the TTET rate, and therefore the photoprotective efficiency. A previous molecular dynamic (MD) investigation of Chl d substituted LHCII revealed only minor reorientation of the bound Chls d,[11] therefore a prevalent role of the differences in the spatial part of the ^{3}Chl d wavefunction in causing the observed differences in the triplet quenching efficiencies is expected."
